# Prevalence and characteristics of cam-type femoroacetabular deformity in 100 hips with symptomatic acetabular dysplasia: a case control study

**DOI:** 10.1186/s13018-014-0093-4

**Published:** 2014-10-10

**Authors:** Takahiro Ida, Yoshinari Nakamura, Tomonobu Hagio, Masatoshi Naito

**Affiliations:** Department of Orthopaedic Surgery, Fukuoka University Faculty of Medicine, 7-45-1 Nanakuma, Jonan-ku, Fukuoka, 814-0180 Japan

**Keywords:** Femoroacetabular impingement, Acetabular dysplasia, Curved periacetabular osteotomy, Alpha angle, Pelvic inclination, Femoral anteversion

## Abstract

**Background:**

Cam-type femoroacetabular deformity in acetabular dysplasia (AD) has not been well clarified. The primary purpose of this study was to determine the prevalence and characteristics of femoroacetabular deformity in symptomatic AD patients.

**Methods:**

We retrospectively reviewed the cases of 86 women (92 hips) and eight men (eight hips) with symptomatic AD. The mean patient age was 37.9 (range, 14–60) years. All participants underwent lateral cross-table and lateral whole-spine radiographic examinations to measure the alpha angle and pelvic tilt. Pelvic computed tomography scans were used to measure femoral anteversion. The patients were classified into two groups: AD only group, containing hips with an alpha angle less than 55°; and AD with cam-type femoroacetabular deformity (AD + cam-type deformity) group, containing hips with an alpha angle greater than or equal to 55°.

**Results:**

Of the patients with AD, 40 hips displayed additional radiographic evidence of cam-type morphology, while 60 hips had exclusive AD morphology. The patients in the AD + cam-type deformity group had significantly increased forward pelvic tilt in the standing position (*p* =0.023) and decreased femoral anteversion (*p* =0.047) compared with the AD only group.

**Conclusions:**

Our data revealed that 40% of patients with AD also had radiographic evidence of cam-type femoroacetabular deformity. Greater forward pelvic tilt in the standing position and decreased femoral anteversion seemed to be associated with the cam-type deformity in these patients. These results indicate the morphological features that are most likely to induce secondary symptoms to developmental hip dysplasia. It is suggested that the symptoms in the AD + cam-type deformity group could arise through femoroacetabular impingement (FAI) after periacetabular osteotomy, because a predisposition was present preoperatively.

## Introduction

It has been increasingly shown that femoroacetabular impingement (FAI) can lead to cartilage damage and labral tears and is therefore a probable cause of progression to premature osteoarthritis in hip joints. FAI is defined as pathological contact between the acetabular rim and the femur, typically at the junction between the anterosuperior femoral head and neck [1-5]. As such, an early diagnosis of FAI is imperative to prevent further damage. FAI is classified as either cam-type or pincer-type based on the underlying anatomical deformity. However, most cases of FAI have both femoral and acetabular involvement [1].

Acetabular dysplasia (AD) is one of the most common causes of hip osteoarthritis and is often associated with deficient coverage of the femoral head [6,7]. AD can induce compensatory anterior inclination of the pelvis to improve approximation of the acetabulum [8]. For patients with symptomatic AD, periacetabular osteotomy is an effective treatment [9-11] to correct structural instability and optimize joint biomechanics. However, it is important to avoid secondary FAI caused by reorientation of the acetabulum and residual deformity of the proximal femur, which, in turn, makes the hip susceptible to femoroacetabular abutment [12]. However, to our knowledge, the exact prevalence and characteristics of cam-type femoroacetabular deformity in AD have been investigated in only a few studies [13,14]. Therefore, the aim of this study was to determine the prevalence and characteristics of cam-type femoroacetabular deformity in patients with symptomatic AD. In particular, we hypothesized that lordotic pelvic and lumbar tilts and residual deformity of the proximal femur are associated with cam-type femoroacetabular deformity in these patients.

## Methods

We retrospectively reviewed 142 consecutive hips in 131 adolescent and adult Japanese patients with symptomatic AD who had undergone curved periacetabular osteotomy (CPO) [11] between May 2009 and June 2012. All patients had been referred to the two senior authors for treatment and provided written informed consent to participate in this study. The study protocol was reviewed and approved by the Institutional Review Board at our hospital. The Fukuoka University Faculty of Medicine institutional review board is approving a usual clinical research by the Department of Orthopaedic Surgery. This study need not be held special ethics committee, and corresponds to a usual clinical research.

The surgical indications for periacetabular osteotomy were as follows: 1) symptomatic AD with a lateral centre-edge (CE) angle [6] of less than 20° or acetabular roof obliquity (ARO) [15] of more than 10°, as measured on anteroposterior radiographs; 2) pain that was tolerable but compromised the patient’s quality of life; 3) partial limitation of daily activities for more than 5 months and 4) improvement of joint congruency on an anteroposterior radiograph in the abducted position.

A total of 37 patients (42 hips) were excluded from this study because of a diagnosis of Legg-Calvé-Perthes disease, poorly taken radiographs with excessive pelvic rotation [16], radiological evidence of advanced osteoarthritis (Tönnis grades 2 and 3) [17] or previous surgical hip intervention. Consequently, we evaluated 94 patients (100 hips), comprising 86 women (92 hips) and eight men (eight hips). The mean patient age was 37.9 (range, 14–60) years at the time of surgery.

Clinical evaluation was based on the Harris hip score (HHS) system [18] and anterior impingement test [5]. The anterior impingement test was performed with the patient in the supine position, and the hip was rotated internally as it was flexed passively to approximately 90° and adducted. This manoeuvre results in approximation of the femoral neck and acetabulum and results in pain among patients with damage to the femoroacetabular rim [19]. The HHS system and anterior impingement test were also performed and body mass index (BMI) was calculated for all patients preoperatively.

**Figure 1:**
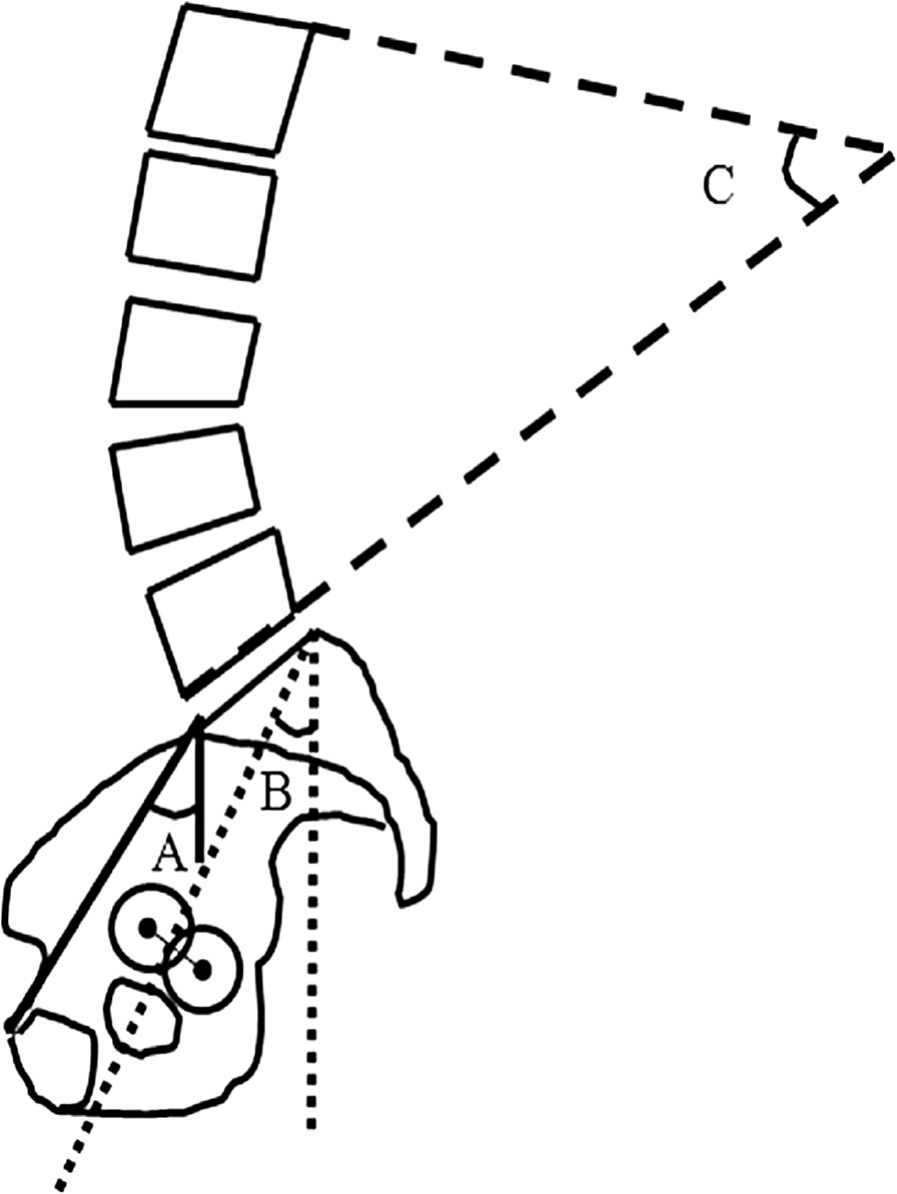
**Diagram showing the radiological indices of lateral whole-spine radiographs in the standing and decubitus positions. (A)** The pelvic inclination angle was formed by the angle between a solid line connecting the promontorium and the upper edge of the symphysis pubis and a vertical line. **(B)** The pelvic angle was formed by the angle between a dotted line extending from the posterior side of the upper edge of the sacrum to the midpoint of a line connecting the central point of the left and right femoral heads and a vertical line. **(C)** The lumbar lordotic angle was formed by the angle between a dashed line extending from L1 to L5.

All patients underwent standardized anteroposterior, cross-table lateral, false-profile lateral and lateral whole-spine radiographs and pelvic computed tomography (CT) of each hip. The anteroposterior radiographic evaluations were performed with neutral rotation of the hip. The measurements included the CE angle, ARO, acetabular head index (AHI) [20], joint-space of the affected hip,and neck-shaft angles (formed by the axis of the femoral neck and the axis of the proximal diaphyseal femur) [21]. The severity of osteoarthritis was classified radiographically using the Tönnis classification system [17] as follows: Grade 0, no sign of osteoarthritis; Grade 1, increased sclerosis, slight joint-space narrowing, no or slight loss of head sphericity; Grade 2, small cysts, moderate joint-space narrowing, moderate loss of head sphericity and Grade 3, large cysts, severe joint-space narrowing, severe deformity of the head. Excessive pelvic rotation was evaluated in terms of the comparative radiographic appearance of the obturator foramen and the position of the sacral midpoint and pubic symphysis. No corrections were made for radiographic magnification. The presence of cam-type femoroacetabular deformity was assessed on cross-table lateral radiographs with 15° of internal rotation of the symptomatic limb by measuring the alpha angle. The alpha angle, originally described in magnetic resonance imaging [22], is increasingly being applied to plain radiography [13] and is formed by the axis of the femoral neck and a line connecting the centre of the femoral head with the start of the asphericity. False-profile lateral radiographic evaluations were performed for measurement of the anterior CE angle [23]. Lateral whole-spine radiographs in the standing and decubitus positions were evaluated by measuring the pelvic inclination [24], pelvic angle [25] and lumbar lordotic angle [26] (Figure 1). If the pelvic inclination and pelvic angle tended to decrease in patients, the pelvic tilt tended toward anterior inclination. The lumbar lordotic angle was defined by the Cobb angle between L1 and L5. If the lumbar lordotic angle tended to increase in patients, the lumbar spine tended toward lordosis.

All pelvic CT scans were acquired by multislice CT (Aquillion 64 DAS; Toshiba Medical Systems, Tochigi, Japan) with patients in a supine position, without any special positioning of the legs. All images were acquired axially at 0.5-mm intervals from the anterior superior iliac spines to below the knee before surgery (120 kV, 155 mA, 0.5-mm slice thickness, 0.5-s rotation time). The pelvic CT scans were evaluated by measuring femoral and acetabular anteversions. For the femoral CT scans, the determination of femoral anteversion was performed systematically [27]. First, a line parallel to the posterior femoral condyles was drawn. Next, a line was drawn through the centre of the femoral neck on the oblique axial images. These images and lines were then superimposed. The angle between the lines was reported as the femoral anteversion (degrees). Acetabular anteversion was defined as the angle made by the line between the anterior and posterior acetabular ridge and a reference line drawn perpendicular to the line between the posterior pelvic margins at the level of the sciatic notch [28]. The acetabular anteversion angle was measured at the level of the centre of the femoral head.

The patients were classified into two groups: AD only group, containing hips with an alpha angle less than 55°; and AD + cam-type deformity group, containing hips with an alpha angle greater than or equal to 55°.

### Statistical analysis

Statistical analyses were performed using the Mann–Whitney *U* test to compare the radiographic parameters, femoral anteversion from pelvic CT scans and HHS between the AD only and AD + cam-type deformity groups. The chi-square test was used to compare the severity of osteoarthritis and measurements of acetabular version and impingement between the two groups. Statistical significance was assumed for *p* values less than 0.05.

For reliability assessment, the alpha angle measurements were repeated by two observers who were blind to the clinical evaluation in 50 randomly selected patients. One observer further evaluated the measurements of the alpha angle, CE angle, femoral anteversion from pelvic CT images and pelvic angle in the standing position in these 50 randomly selected patients. These measurements were repeated three times on different occasions at intervals of not less than 2 weeks. Subsequently, the interobserver and intraobserver reliability coefficients were evaluated by intraclass correlation coefficient testing.

## Results

The cohort included eight male (eight hips) and 86 female (92 hips) patients (age range, 14–60 years). The total of 100 hips were classified into AD only (*n* =60) and AD + cam-type deformity (*n* =40) groups (Table 1). There were no significant differences in preoperative HHS and BMI between the groups. However, there were significant differences in the preoperative positive anterior impingement test between the groups (*p* =0.007).

**Table 1:** **Baseline characteristics of the patients in the acetabular dysplasia (AD) only and AD + cam-type deformity groups**

**Parameters**	**AD only**	**AD + cam-type deformity**	***p*** **value**
No. of hips	60	40	-
Sex (men:women) (no. of hips)	3:57	5:35	0.176
Mean (SD; range) age (years)	37.2 (13.0; 14 to 60)	39.0 (11.2; 15 to 59)	0.497
Tönnis grade (0:1) (no. of hips)	23:37	10:30	0.165
Mean (SD; range) HHS	76.6 (10.3; 47 to 96)	76.3 (11.0; 43 to 96)	0.83
Mean (SD; range) BMI (kg/m^2^)	22.1 (3.0; 15.9 to 32.4)	22.0 (3.1; 15.9 to 31.4)	0.85
Anterior impingement test (positive:negative) (no. of hips)	25:35	30:10	0.007^*^

*p <0.05 (chi-square test) for the difference between the groups. *HHS* Harris hip score; *BMI* body mass index; *SD* standard deviation.

The preoperative radiographic evaluations are shown in Tables 2 and 3. There were no significant differences in the mean values for any of the preoperative radiographic parameters on anteroposterior and false-profile lateral radiographs between the groups. In particular, the absence of significant differences in the anterior and lateral CE angles suggested that the grade of coverage of the femoral head in AD was not significantly different between the groups. However, significant differences were found for preoperative pelvic inclination and pelvic angle in the standing position between the groups (*p* =0.023 and *p* =0.006, respectively). Patients in the AD + cam-type deformity group had significantly more forward pelvic tilt in the standing position than patients in the AD only group. There were no significant differences in the lumbar lordotic angles on lateral whole-spine radiographs in the standing and lateral decubitus positions.

**Table 2:** **Preoperative radiographic evaluations in the acetabular dysplasia (AD) only and AD + cam-type deformity groups**

**Parameters**	**AD only (** ***n*** **=60)**	**AD + cam-type deformity (** ***n*** **=40)**	***p*** **value**
	**Mean (SD; range)**	**Mean (SD; range)**	
Alpha angle (degrees)	40.2 (6.7; 26.7 to 53.0)	73.5 (14.4; 56.0 to 118.0)	<0.001^*^
Lateral centre-edge angle (degrees)	11.3 (7.1; −10.0 to 22.8)	11.4 (6.7; −7.0 to 21.7)	0.975
Acetabular roof obliquity (degrees)	17.9 (7.3; 5.1 to 36.3)	19.1 (9.8; 5 to 31.9)	0.632
Acetabular head index (%)	65.8 (8.9; 39.8 to 83.6)	64.9 (8.7; 40.8 to 80.2)	0.586
Joint space (mm)	4.2 (1.1; 2.2 to 6.3)	4.1 (1.0; 2.5 to 6.0)	0.733
Neck-shaft angle (degrees)	133.9 (5.6; 125.2 to 146.1)	134.7 (6.5; 125.3 to 154)	0.688
Anterior centre-edge angle (degrees)	12.9 (11.8; −14.5 to 30.7)	12.5 (12.0; −14.7 to 33.7)	0.563

*p <0.05 (Mann–Whitney *U* test) for the difference between the groups. *SD* standard deviation.

**Table 3:** **Lateral whole-spine radiographic evaluations in the acetabular dysplasia (AD) only and AD + cam-type deformity groups**

**Parameters**	**AD only (** ***n*** **=60)**	**AD + cam-type deformity (** ***n*** **=40)**	***p*** **value**
	**Mean (SD; range)**	**Mean (SD; range)**	
Pelvic inclination angle (degrees)			
Standing position	29.9 (6.4; 17.3 to 44.5)	26.1 (8.1; 12.7 to 39.8)	0.023^*^
Decubitus position	22.6 (7.8; 4.3 to 38.4)	22.1 (9.4; 3.9 to 41.5)	0.746
Pelvic angle (degrees)			
Standing position	22.3 (6.2; 9.4 to 32.8)	18.3 (7.7; 5.5 to 36.3)	0.006^*^
Decubitus position	14.2 (7.4; −3.4 to 31.8)	13.1(9.3; −3.5 to 27.7)	0.595
Lumbar lordotic angle (degrees)			
Standing position	38.1 (13.0; 0.5 to 69.3)	38.1 (12.8; 13.7 to 63.7)	0.847
Decubitus position	36.9 (13.4; −2.9 to 63.6)	36.0 (13.3; 6.8 to 36.3)	0.833

*p <0.05 (Mann–Whitney *U* test) for the difference between the groups. *SD* standard deviation.

The mean femoral anteversion value, as measured on pelvic CT scans, was 20.8° (range, 0.5°–47.4°). The AD + cam-type deformity group had significantly decreased femoral anteversion compared with the AD only group (*p* =0.047; Table 4). Preoperative acetabular anteversion was not significantly different between the groups.

**Table 4:** **Femoral and acetabular anteversions on computed tomography images in the acetabular dysplasia (AD) only and AD + cam-type deformity groups**

**Parameters**	**AD only (** ***n*** **=60)**	**AD + cam-type deformity (** ***n*** **=40)**	***p*** **value**
	**Mean (SD; range)**	**Mean (SD; range)**	
Femoral anteversion (degrees)	22.4 (10.2; 0.5 to 47.4)	18.5 (10.6; 2.5 to 46.9)	0.047^*^
Acetabular anteversion (degrees)	21.8 (6.0; 11.2 to 34.8)	21.0 (6.1; 8.1 to 32.2)	0.786

*p <0.05 (Mann–Whitney *U* test) for the difference between the groups. *SD* standard deviation.

The interobserver reliability coefficient for measurements of the alpha angle was 0.85. The intraobserver reliability coefficients for measurements of the alpha angle, CE angle and femoral anteversion on pelvic CT scans and pelvic angle in the standing position were 0.98, 0.93, 0.96 and 0.97, respectively.

## Discussion

Femoroacetabular deformities associated with AD have been documented in a few reports [13,14]. Paliobeis et al. [14] reported that 47% of patients with FAI also had radiographic evidence of dysplasia. Clohisy et al. [13] reported that 73.1% of dysplastic hips had an abnormal head-neck ratio or alpha angle and 72% were judged to have an aspheric femoral head in their analysis of the femoral head-neck junction in symptomatic AD. However, Paliobeis et al. did not show the prevalence of only cam-type femoroacetabular deformity in AD and included pincer and combined types of FAI in AD [14]. Furthermore, Clohisy et al. [13] included patients who had at least one prior osteotomy and defined proximal femoral abnormalities as either an alpha angle of greater than 50° or femoral head-neck offset of less than 9 mm, as described by Eijer et al. [29]. In our study, we used the alpha angle on cross-table lateral radiographs to determine cam-type femoroacetabular deformity. Although the validity of applying these definitions across imaging modalities remains questionable, an alpha angle greater than 55° has been considered to reflect a characteristic of cam-type femoroacetabular deformity by other researchers [22,28,30,31]. Although there were no significant differences in the mean patient age, sex, severity of osteoarthritis or severity of AD (i.e. anterior and lateral CE angles, ARO and AHI) between the groups, we found that 40.0% of patients with AD had additional radiographic evidence of cam-type femoroacetabular deformity.

In our study, the AD + cam-type deformity group had significantly more forward pelvic tilt in the standing position (*p* =0.023) and decreased femoral anteversion (*p* =0.047) compared with the AD only group. However, no significant differences were observed in the lumbar lordotic angles. Other studies examined the relationship between the hip joint or pelvis and the lumbar spine, largely in terms of the differences in lumbar spine alignment [8,32]. In particular, the studies found that osteoarthritis of the subluxated hip joint induced compensatory anterior inclination of the pelvis to improve approximation of the acetabulum. Conversely, Okuda et al. [24] reported that patients with pre-arthritic/early osteoarthritis of the hip joint tended to have anterior inclination of the pelvis compared with healthy volunteers of a similar age, but there was no significant difference in the lumbar lordotic angle between the groups. This probably arose because the sacroiliac joint compensated to maintain the alignment of the lumbar spine. It has also been shown that abnormal anteversion of the femoral neck is related to several disease processes [21,33]. As previously reported, the degree of femoral anteversion in AD is significantly larger than that in normal hips [34,35]. Additionally, Botser et al. [27] found a significant correlation between femoral anteversion and the range of internal rotation of the hip as well as a relevant correlation between cam-type impingement and a lower degree of anteversion. Our results are in accordance with two of these previous reports [24,27]. In other words, these results indicate the morphological features that are most likely to induce secondary symptoms to developmental hip dysplasia. Audenaert et al. [28] reported that cam size, acetabular coverage and femoral anteversion were the main determinants for predicting differences in internal rotation during impingement testing. Accordingly, in our study, the AD + cam-type deformity group had a significantly higher ratio in the positive anterior impingement test than the AD only group. Thus, the anterior impingement test may be a valid method to determine the prevalence of cam-type femoroacetabular deformity in AD.

**Figure 2:**
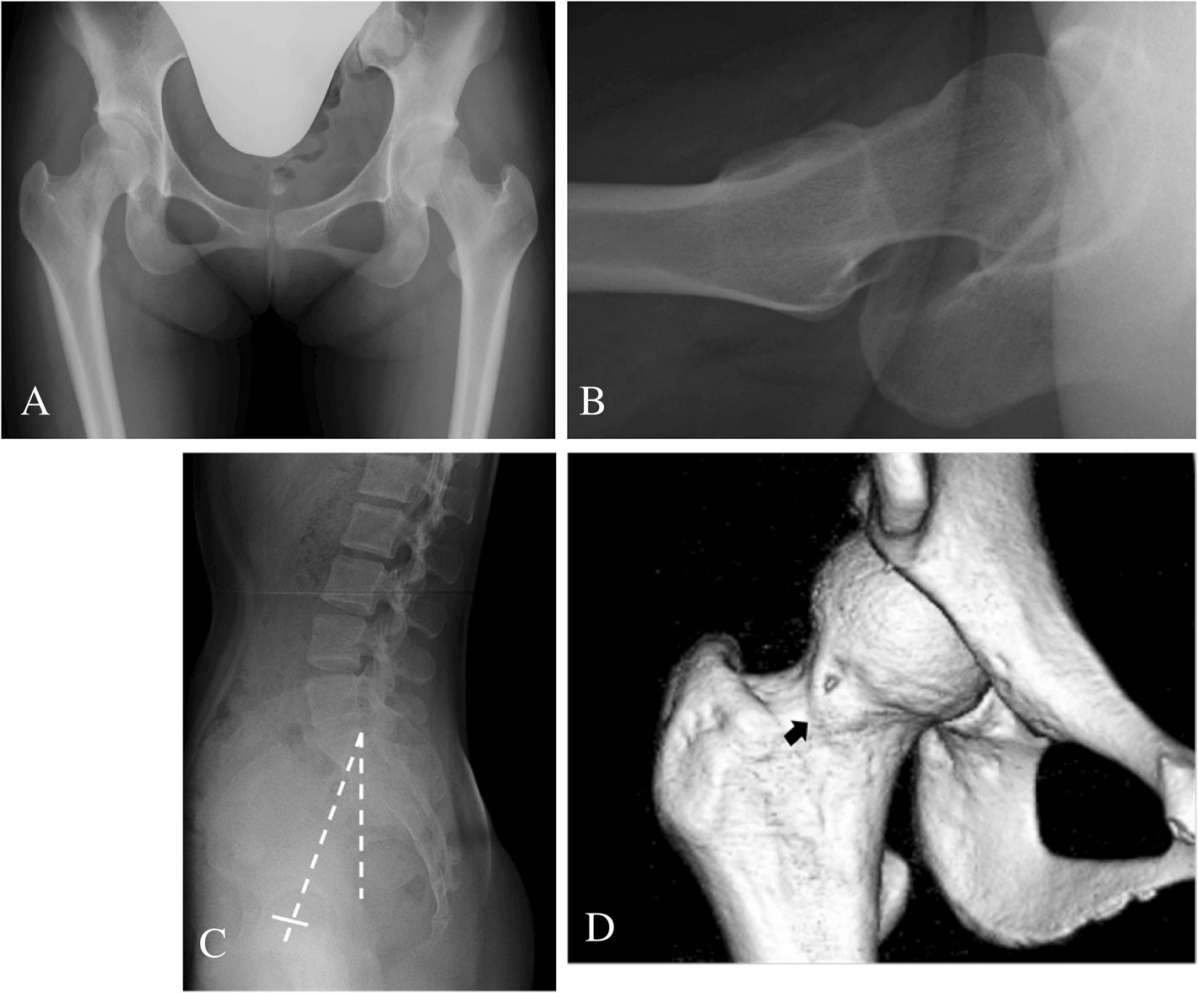
**Examples of preoperative radiographs and three-dimensional computed tomography (CT).** A 23-year-old woman with right hip pain presented with acetabular dysplasia and a non-spherical femoral head-neck junction. Radiographs and three-dimensional CT images were taken prior to curved periacetabular osteotomy. **(A)** The centre-edge angle and acetabular roof obliquity were 19.0° and 13.0°, respectively. **(B)** The alpha angle was 61°. **(C)** The pelvic angle was 16.4° in the standing position, as indicated by the dashed lines. **(D)** The arrow indicates cam-type femoroacetabular deformity on a three-dimensional CT image.

**Figure 3:**
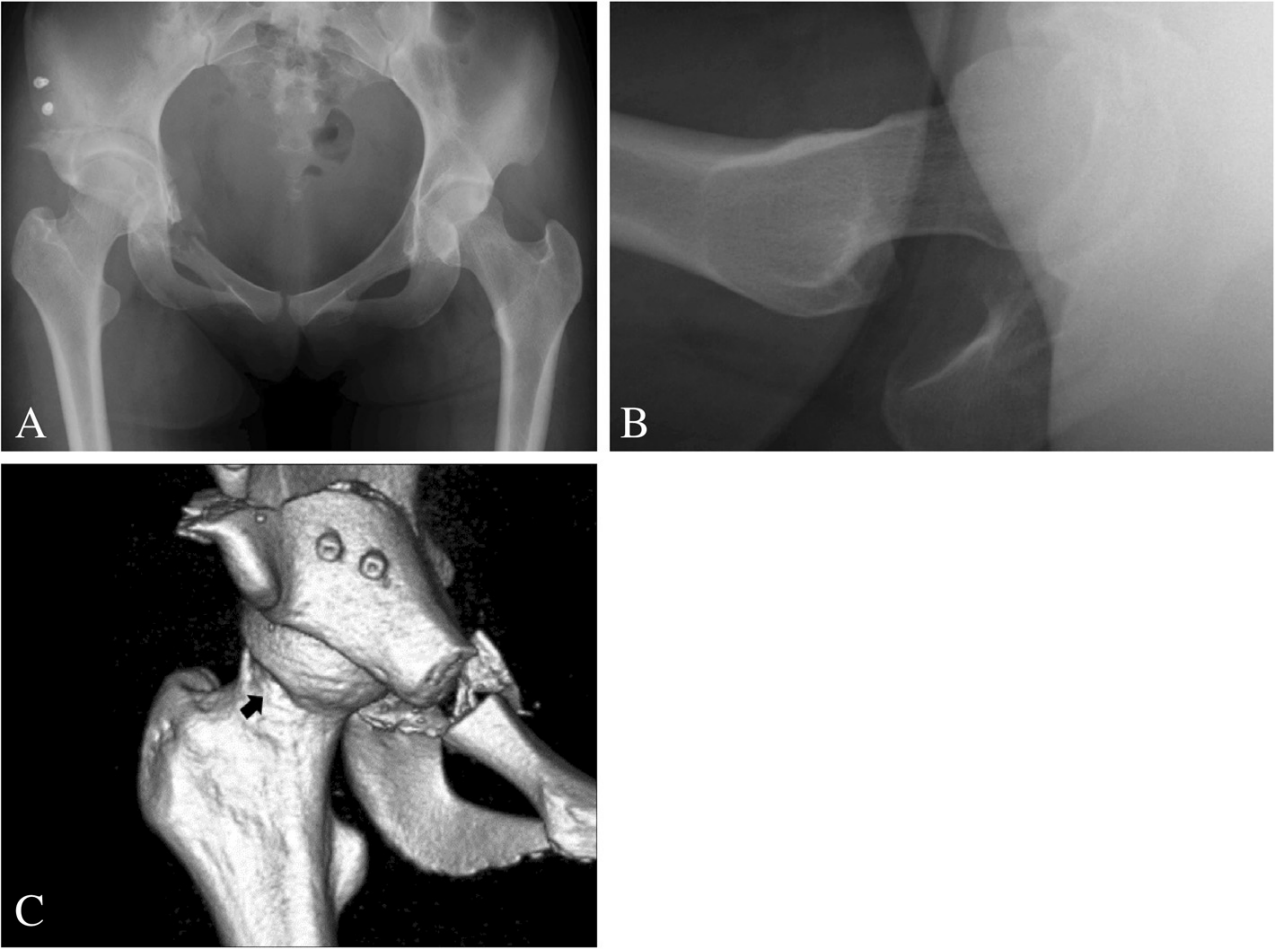
**Example of postoperative radiographs and three-dimensional CT.** A 23-year-old female presented with cam-type femoroacetabular deformity and acetabular hip dysplasia. Radiographs and three-dimensional CT images were taken 1 week after curved periacetabular osteotomy. **(A)** The centre-edge angle and acetabular roof obliquity were 33.0° and 0°, respectively. **(B)** The alpha angle was 40.0°. **(C)** The arrow indicates the spherical junction of the femoral head-neck on a three-dimensional CT image after curved periacetabular osteotomy and osteochondroplasty.

A few studies have examined whether development of secondary FAI after acetabular reorientation is one of the major causes of clinical failure [12,13,36,37]. Troelsen et al. [36] reported an 81.6% survivorship rate at a mean of 9.2 years after periacetabular osteotomy, with 14% of hips requiring total hip replacement at a mean of 6.8 years. Of the surviving hips, 34% had groin pain, 25% had clicking or locking and 18% had a positive impingement test. Despite the overall good results, these symptoms raise the issue of residual FAI as a potential contributing factor to clinical failure. Nassif et al. [37] reported that periacetabular osteotomy provides reliable intermediate and long-term results for patients with symptomatic acetabular dysplasia. However, there is increasing evidence that secondary FAI may be a cause of ongoing clinical symptoms. It seems plausible that the presence of femoral head deformities and forward pelvic tilt can still be neutralized by the global undercoverage of the femoral head that is inherent to AD. Therefore, it is suggested that periacetabular osteotomy in the setting of femoral head deformities and forward pelvic tilt has the potential to provoke secondary FAI. Furthermore, Nassif et al. [37] reported the results of femoral head-neck junction osteochondroplasty performed concurrently with periacetabular osteotomy for the treatment of symptomatic AD associated with femoral head-neck junction deformity. This combined procedure provided effective correction of associated femoral head-neck deformities and produced similar early functional outcomes to isolated periacetabular osteotomy. We have also been performing CPO in conjunction with osteochondroplasty for the treatment of AD associated with FAI since 2006 [38] (Figures 2 and 3). Although it usually takes about 15 min longer than isolated periacetabular osteotomy, the combined procedure has been providing effective correction of both acetabular dysplasia and associated femoral head-neck deformities without any increase in the complication rate.

We acknowledge several limitations of this study. First, there was no significant difference in the preoperative HHS between the groups. However, the clinical outcomes of cam-type deformity in AD following periacetabular osteotomy without osteochondroplasty are still undefined. In a previous study, satisfactory results were obtained clinically and radiographically after periacetabular osteotomy in most of the patients without paying attention to the femoral head-neck junction [11]. However, in hips with an aspheric femoral head-neck junction, secondary FAI can be problematic after periacetabular osteotomy [12]. Therefore, since 2009, we have performed periacetabular osteotomy and osteochondroplasty during open or arthroscopic surgeries for the treatment of symptomatic AD in adolescent and adult patients with cam-type femoroacetabular deformity (Figure 3). Second, the causes of increased forward pelvic tilt in the standing position or decreased femoral anteversion in AD patients are still undefined. As previously described, the pelvic inclination is regulated by the muscles around the hip [39]. Akiyama et al. [35] outlined that variability in femoral anteversion is considered to exist from the early stages of life. This increased anatomical variability may lead to controversy in relating femoral anteversion to other anatomical measurements. Third, we cannot definitively state the exact location on the femoral head-neck junction through the use of cross-table lateral radiographs with 15° of internal rotation of the symptomatic limb. However, we considered it reasonable to use this method, because Meyer et al. [40] reported that high-sensitivity cross-table lateral radiographs could be obtained with the leg at approximately 15° of internal rotation. Additionally, the cross-table lateral radiograph is a cost-efficient, albeit slightly inaccurate, way to measure offset alpha angles in the clinical setting [41]. Fourth, the prevalence of cam-type deformity in AD may be affected by sex or ethnic differences. As previously reported, radiographic features suggestive of cam-type FAI are quite common in healthy young adults, especially males [42]. In our study, there were no significant differences in the sex distribution between the groups. However, sex differences cannot be excluded in this study because the number of men in our cohort was small. Additionally, because the patients in this study were all Japanese, it is possible that there is an ethnicity bias in our study.

## Conclusions

Our data revealed that 40.0% of patients with AD also had radiographic evidence of cam-type femoroacetabular deformity. A greater forward pelvic tilt and decreased femoral anteversion appeared to be associated with the cam-type femoroacetabular deformity in these patients. These results indicate the morphological features that are most likely to induce secondary symptoms to developmental hip dysplasia. Therefore, it is suggested that the symptoms in the AD + cam-type deformity group could arise through FAI after periacetabular osteotomy, because a predisposition was present preoperatively.

## Abbreviations

FAI: Femoroacetabular impingement
AD: Acetabular dysplasia
CE: Centre-edge
ARO: Acetabular roof obliquity
HHS: Harris hip score
BMI: Body mass index
CT: Computed tomography
AHI: Acetabular head index
